# miR-520c-3p regulates IL-1β-stimulated human chondrocyte apoptosis and cartilage degradation by targeting GAS2

**DOI:** 10.1186/s13018-021-02466-7

**Published:** 2021-05-29

**Authors:** Le Peng, Ming Deng, Yonggang Ma, Wei Hu, Fan Liang

**Affiliations:** Department of Orthopaedics, Renmin Hospital of Wuhan University, No. 238, Jiefang Road, Wuchang District, Wuhan City, 430060 Hubei Province China

**Keywords:** miR-520c-3p, GAS2, Osteoarthritis, Apoptosis

## Abstract

**Background:**

MicroRNAs (miRNAs) have been shown to be associated with osteoarthritis (OA) progression. This study aimed to explore the role of miR-520c-3p in OA progression.

**Methods:**

Expression levels of miR-520c-3p and Growth arrest-specific 2 (GAS2) were detected using quantitative real-time PCR. The proliferation and apoptosis of cells were measured using cell counting kit 8 (CCK8) assay and flow cytometry. Furthermore, the protein levels of apoptosis-related markers, extracellular degradation markers, inflammatory response markers, and GAS2 were tested using quantitative real-time polymerase chain reaction (RT-PCR) and western blot (WB) analysis. In addition, the interaction between miR-520c-3p and GAS2 was examined using dual luciferase reporter assay.

**Results:**

GAS2 was highly expressed, and miR-520c-3p was lowly expressed in OA cartilage tissues. miR-520c-3p could promote the proliferation and inhibit the apoptosis and inflammation of OA chondrocytes. miR-520c-3p could be sponged by GAS2, and its inhibitor could reverse the regulation of GAS2 on the biological functions of OA chondrocytes. GAS2 was a target of miR-520c-3p, which was identified by bioinformatic analysis and dual-luciferase reporter assay. Overexpression of GAS2 could inhibit the proliferation and promoted the apoptosis and inflammation of OA chondrocytes.

**Conclusion:**

Our data showed that miR-520c-3p might regulate the GAS2 to inhibit the progression of OA.

## Background

Osteoarthritis (OA) is degenerative disease and is a major cause of pain and physical disability [1]. Approximately 15% of the global population suffer from OA [2]. Genetics, aging, obesity, and mechanical stress are the primary causes of OA [3]. Clinical therapy for OA includes nonsteroidal anti-inflammatory drugs (NSAIDs), platelet-rich plasma (PRP), or hyaluronic acid (HA) injections [4, 5]. However, currently no ideal treatment strategies have been developed to prevent the OA progression [6]. Chondrocytes are responsible for normal maintenance and remodeling of articular cartilage and extracellular matrix [7]. The inflammatory mediator interleukin 1 beta (IL-1β) or tumor necrosis factor-α (TNF-α) promotes inflammation response, extracellular matrix degradation, and chondrocyte apoptosis and finally causes cartilage damage [8].

Although there are numerous factors involved in the development of OA, IL-1β has been reported to play a dominant role in the pathogenesis of OA. IL-1β can significantly increase cartilage damage and thus used to mimic OA in vitro studies [9]. Therefore, fully elucidated, the molecular mechanisms of OA are imperative for the development of novel therapeutic strategies.

MicroRNAs (miRNAs), small noncoding RNAs that function in the posttranscriptional regulation of genes, are involved in a number of physiological functions and disease processes, including OA [10]. In previous studies, it was found that the occurrence and development of OA are accompanied by changes in various miRNAs. Zhang et al. [11] found that miR-132 regulates the development of OA through modulation of PTEN/PI3K/AKT signaling pathway. Zhang et al. [12] revealed that miR-130b downregulation potentiates chondrogenic differentiation of bone marrow mesenchymal stem cells by targeting SOX9. However, the pathogenesis of OA is still far from being fully understood. miR-520c-3p has been found to mediate osteosarcoma progression [13], vascular endothelium dysfunction [14], and cholangiocarcinoma progression [15]. We conducted a bioinformatic analysis and found that miR-520c-3p was upregulated in OA-affected tissue than normal cartilage tissue. Accordingly, it may be speculated that miR-520c-3p plays an important role in the initiation and progression of OA.

Mechanism of action of miRNA was that miRNA can affect target gene expression through a 3′-untranslated region binding manner. Growth arrest-specific 2 (GAS2) gene was originally identified in a genetic screen of murine fibroblasts that were cultured under growth arrest conditions [16]. Recently, researcher found that GAS2 was involved in the regulation of apoptosis and chondrogenesis in the developing mouse limb [17]. Through bioinformatic analysis, we found that miR-520c-3p has putative binding sites with GAS2.

In this study, we found that the overexpression of GAS2 was able to reverse the protective effect of miR-520c-3p on IL-1β-induced chondrocyte apoptosis. These findings may substantiate miR-520c-3p as a new therapeutic target for the treatment of OA.

## Methods

### Clinical samples and cell culture

Cartilage tissue samples were collected from patients (*n*=20) with end-stage symptomatic hip OA and patients (*n*=20) without OA histories by total hip arthroplasty at Renmin Hospital of Wuhan University in this study, which was endowed by the Ethics Committee of Renmin Hospital of Wuhan University. And written informed consent was signed by all tissue donors involved in the present research.

Under a humidified incubator with 5% CO_2_ at 37°C, human chondrocyte (American Type Culture Collection, Manassas, VA, USA) were allowed to grow in Dulbecco’s modified Eagle’s medium (DMEM; Gibco, Carlsbad, CA, USA) with 10% fetal bovine serum (FBS; Gibco) and 0.1 mg/mL G-418 (Sigma-Aldrich, St. Louis, MO, USA). For the establishment of the cell model of OA, chondrocytes were induced with 0, 5, 10, and 15 ng/mL of IL-1β (Sigma-Aldrich) for multiple time points (0 h, 6 h, 12 h, 24 h, and 48 h). Besides, 10 ng/mL of IL-1β treatment for 24 h was selected for further functional experiments.

### Real-time quantitative polymerase chain reaction (RT-qPCR)

According to the producer’s instructions of TRIzol reagent (Invitrogen, Carlsbad, CA, USA), total RNAs from tissues and cells were extracted, followed by reverse transcription using PrimeScript RT Reagent Kit (TaKaRa, Tokyo, Japan). On Thermal Cycler CFX6 System (Bio-Rad, Hercules, CA, USA), RT-qPCR reaction was performed referring to the operation manual SYBR Green PCR Kit (TaKaRa). After normalization with GAPDH for Cas-3, Bcl-2, MMP-13, COL2A1, IL-6 and IL-8 and U6 for miR-520c-3p, the levels of gene were calculated by the 2^−ΔΔCt^ method. The specific primers were displayed as: miR-520c-3p: 5′-GGAAGTGGTGGCTATGAGTCAG-3′ (sense), 5′-TGTCAATTTGAAACTTAAAAAGCAG-3′ (antisense); Cas-3: 5′-CTGCCACAGAACCAGTTCCC-3′ (sense), 5′-CTGTGACACGCCTGTTTGGG-3′ (antisense); Bcl-2: 5′-GCATCGTTCCTTCAAGCCGATCT-3′ (sense), 5′-TGGGTGAGTCGTTCGG-3′ (antisense); MMP-13: 5′-ATACGTGGATTGAGGACCACT-3′ (sense), 5′-TCCAATGTCAAGTAGCGGTTG-3′ (antisense); COL2A1: 5′-CTCGCTTCGGCAGCACA-3′ (sense), 5′-CTGCCTTCCTGCACCAAGTA-3′ (antisense); IL-6: 5′-AUTTCGCAGTTGCAT-3′ (sense), 5′-TGGAACAGCACGGATTTGGA-3′ (antisense); IL-8: 5′-GGCAATGCCATTCTACAGATACT-3′ (sense), R 5′-CAACCGAGAAATCCAGCACCT-3′ (antisense); GAS2: F 5′-TGGAACAGCACGGATTTGGA-3′ (sense), R 5′-CTGCCTTCCTGCACCAAGTA-3′ (antisense); GAPDH: 5′-GTCAACGGATTTGGTCTGTATT-3′ (sense), 5′-AGTCTTCTGGGTGGCAGTGAT-3′ (antisense).

### Cell viability assay

After treatment with IL-1β, transfected and un-transfected chondrocytes (5 ×10^3^ cells/well) were introduced into 96-well plates, followed by incubation for 24 h. Whereafter, 10 μL CCK-8 (Dojindo, Kumamoto, Japan) was added into each well for another 4 h. Based on the user’s guidebook of a microplate reader (Bio-Rad, Hercules, CA, USA), the absorbance at 450 nm was determined.

### Western blot assay

For total protein preparation, chondrocytes were treated with pre-cold RIPA buffer (Beyotime, Nantong, China), followed by quantification using bicinchoninic acid (BCA) Protein Assay Kit (Solarbio, Beijing, China). For immunoblotting, the protein samples (50 μg) were separated by 10% SDS-PAGE and electrophoretically transfected to nitrocellulose membranes (Millipore, New York, NY, USA). And then, the membranes were subjected to hybridize with primary antibodies: cleaved cas-3 (1:1000, Abcam, Cambridge, MA, USA), Bcl-2 (1:1000, Abcam, Cambridge, MA, USA), MMP-13 (1:1000, Abcam, Cambridge, MA, USA), COL2A1 (1:1000, Abcam, Cambridge, MA, USA), IL-6 (1:1000, Abcam, Cambridge, MA, USA), IL-8 (1:1000, Abcam, Cambridge, MA, USA), and GAPDH (1:1000, Abcam, Cambridge, MA, USA) at 4°C overnight. After incubation with secondary antibody (1:10,000, Abcam, Cambridge, MA, USA) for 2 h, the detection of protein bands was performed using ECL detection system (GE Healthcare, Piscataway, NJ, USA).

### Cell transfection

For miR-520c-3p overexpression, chondrocytes were transfected with 50 nM of miR-520c-3p overexpression plasmid (miR-520c-3p, RiboBio, Guangzhou, China) and its negative control NC mimic (RiboBio). For GAS2 overexpression, the sequence of GAS2 was introduced into pcDNA empty vector (vector, Invitrogen, a negative control) to obtain pcDNA-GAS2 (GAS2) vector, and then, these vectors (200 ng) were transfected into chondrocytes. In this study, all transfection was employed according to the instructions Lipofectamine 3000 (Invitrogen) for 48 h.

### Cell apoptosis assay

Generally, treated chondrocytes were collected and washed, followed by re-suspending Binding Buffer. After incubation with 5 μL Annexin (V-fluorescein isothiocyanate) V-FITC and 10 μL propidium iodide (PI) (Roche, Indianapolis, IN, USA) for 15 min, the measurement of the apoptosis rate was conducted following the operation manual of a FACS Calibur (BD Bioscience, San Jose, CA, USA).

### Dual-luciferase reporter assay

In this assay, the binding relationship between miR-520c-3p and GAS2 was predicted by TargetScan software, as verified by a dual-luciferase reporter assay. In short, the GAS2 wild-type (WT) reporter vector (WT- GAS2) 3′ untranslated region (3′UTR) were generated by inserting the fragment sequence of GAS2 3′UTR with the putative binding site of miR-520c-3p into pmirGLO plasmids (Promega, Madison, WI, USA). Analogously, the corresponding mutant fragments of GAS2 3′UTR were applied to build the vectors Mutant (MUT)-GAS2 3′UTR. Whereafter, the constructed vectors were transfected into chondrocytes along with miR-520c-3p or NC mimic, in line with the user’s guidebook of Lipofectamine 3000 reagent (Invitrogen). At length, the assessment of the luciferase activities was carried out using a dual-luciferase reporter assay kit (Promega) after transfection for 48 h.

### Statistical analysis

SPSS statistical software (version 20.0; IBM SPSS, Armonk, NY, USA) was used to conduct the statistical analysis. All data were presented as mean ± standard deviation from 3 independent experiments. Results from three or more groups were analyzed by one-way analysis of variance followed by Tukey’s multiple comparison test. Statistical analysis was performed by Student’s *t*-test when two groups were analyzed. *P* < 0.05 was considered statistically significant.

## Results

### MiR-520c-3p was upregulated in OA-affected cartilage and inhibited the IL-1β-induced chondrocytes’ apoptosis

**Fig. 1:**
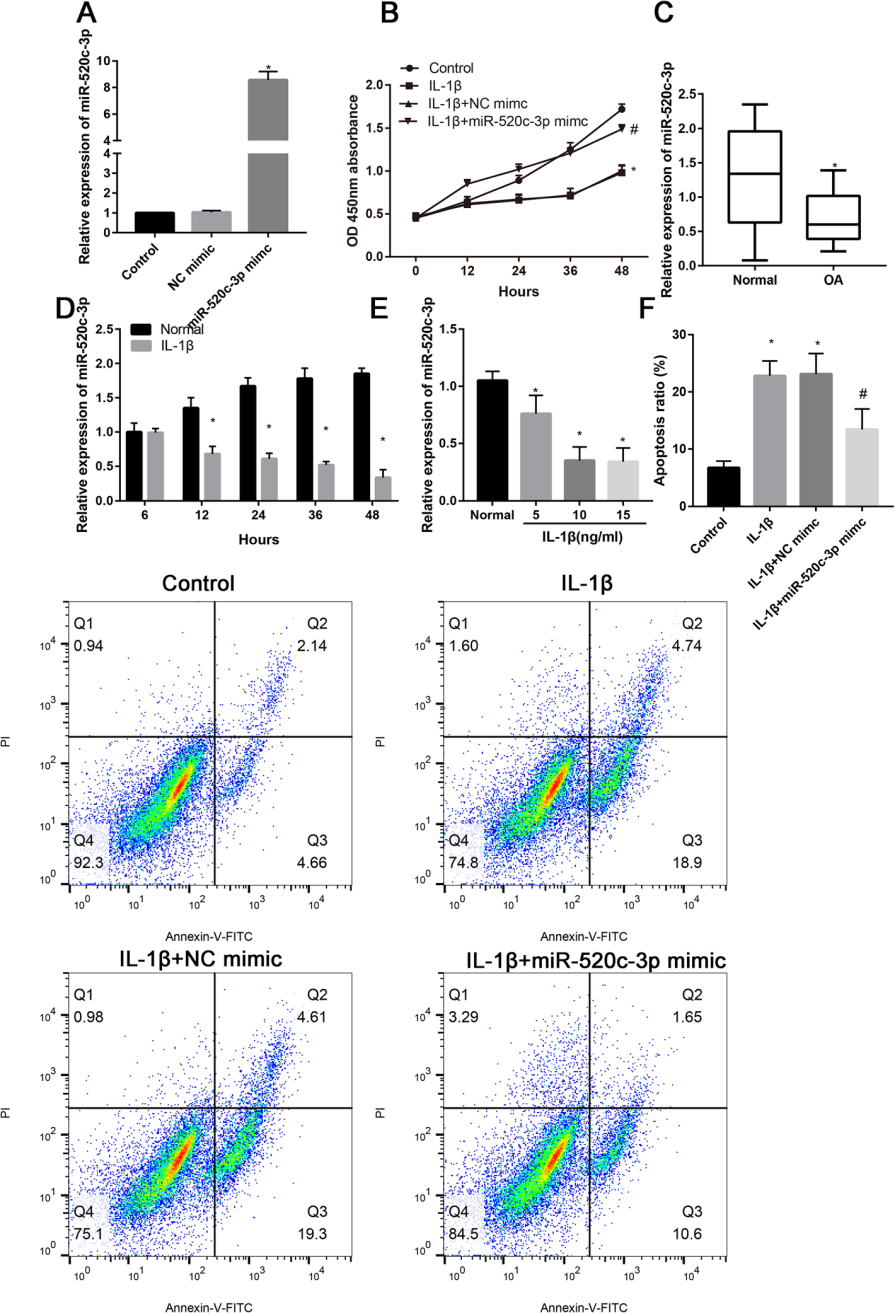
Overexpression of miR-520c-3p alleviated IL-1β-stimulated chondrocyte apoptosis. **a** Relative miR-520c-3p expression in control, IL-1β, and IL-1β+miR-520c-3p mimic, *P < 0.05 versus control or NC mimic groups. **b** Chondrocyte viability at different time points (0 h, 12 h, 24 h, 36 h, and 48 h) in control, IL-1β-stimulated, and NC mimic- or miR-520c-3p mimic-transfected cells, * *P*<0.05 versus control group, #*P*<0.05 versus IL-1β or IL-1β +NC mimic. **c** Relative expression of miR-520c-3p in normal and OA-affected cartilage, *P<0.05 versus normal group. **d** Relative expression of miR-520c-3p in normal and IL-1β-treated chondrocytes at different hours (6, 12, 24, 36, and 48 h), *P<0.05 versus normal group. **e** Relative miR-520c-3p expression in control, IL-1β, IL-1β+NC mimic, and IL-1β+miR-520c-3p mimic groups, *P<0.05 versus normal group. **f** Annexin V and PI double staining was performed to identify the apoptosis ratio in the control, IL-1β, IL-1β + NC mimic, and IL-1β + miR-520c-3p mimic groups by flow cytometry. *P<0.05 versus control group, #*P*<0.05 versus IL-1β or IL-1β +NC mimic

In order to identify the function of miR-520c-3p for the chondrocytes, we constructed miR-520c-3p mimic and the transfection efficacy was identified by RT-PCR. After transfected with miR-520c-3p mimic, the miR-520c-3p expression increased about 8-fold than NC mimic (Fig. 1a). Then, CCK-8 assay was performed to identify the role of miR-520c-3p for cell viability. Results suggested that IL-1β significantly decreased the cell viability at 12, 24, 36, and 48 h (Fig. 1b). The partial inhibition of cell viability by IL-1β could be reversed by addition of miR-520c-3p at 36 and 48 h (Fig. 1b). To identify the relative expression of miR-520c-3p between normal and OA-affected cartilage, RT-PCR was performed. We found that miR-520c-3p was significantly downregulated in OA-affected cartilage (Fig. 1c, *P*<0.05). Moreover, the relative expression of miR-520c-3p was significantly downregulated after treatment with IL-1β (10 ng/ml) at 12, 24, 36, and 48 h (Fig. 1d, *P*<0.05). Then, different concentration of IL-1β were treated with chondrocytes; we found that a significantly downregulated miR-520c-3p was observed after IL-1β concentration increased (Fig. 1e, *P*<0.05). However, the expression did not change significantly when the IL-1β concentration was further increased to 15 ng/ml (Fig. 1e, *P*>0.05). In addition, cell apoptosis rates were detected using flow cytometry. As shown in Fig. 1f, IL-1β resulted in significant increase in apoptosis incidence (∼28%) than control group. While miR-520c-3p mimic significantly inhibited IL-1β-induced apoptosis (Fig. 1f).

### MiR-520c-3p significantly downregulated apoptotic-related, inflammation response markers and upregulated extracellular matrix-associated markers

**Fig. 2:**
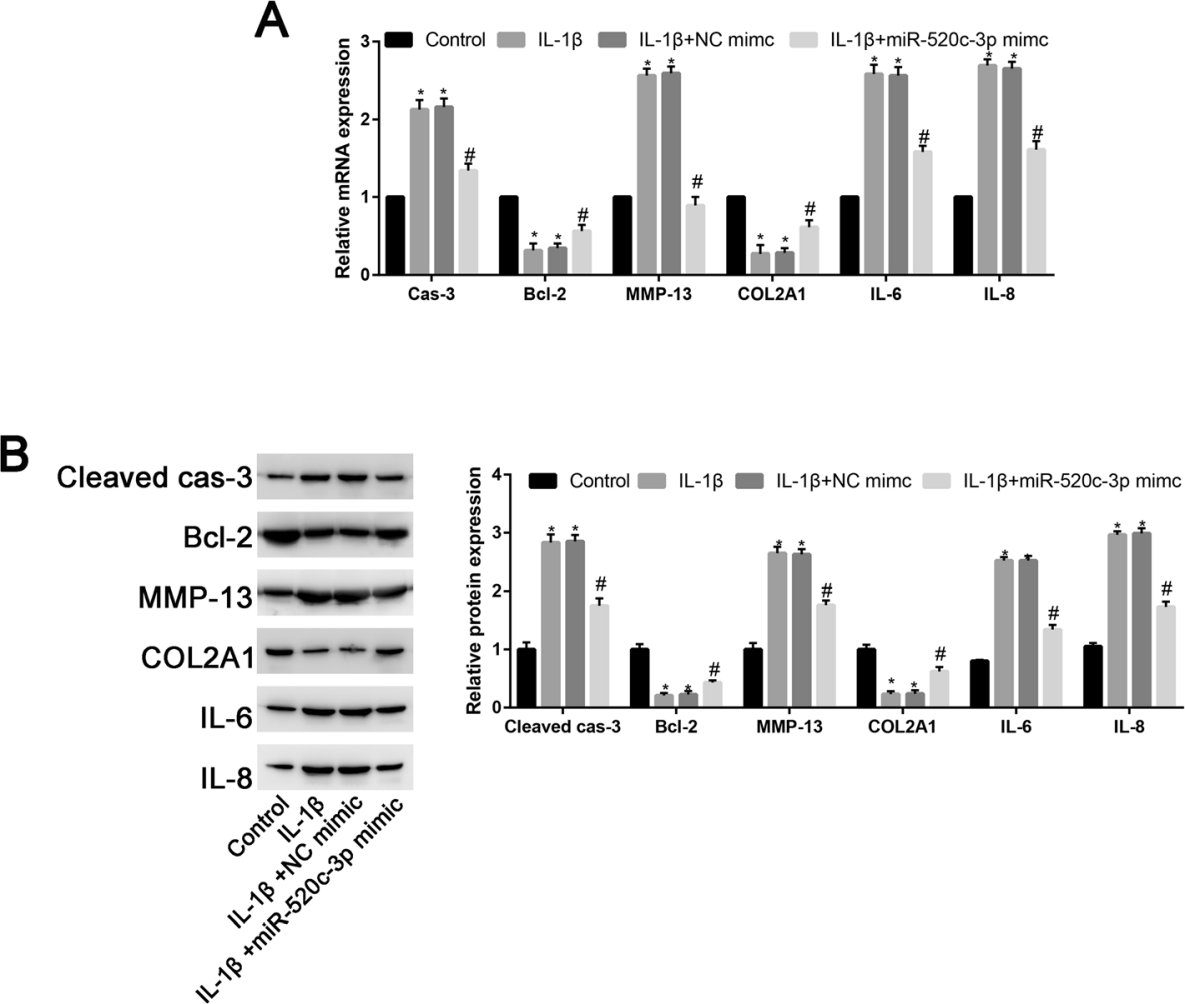
Protective role miR-520c-3p in alleviating IL-1β induced the apoptosis, inflammation response, and matrix degradation of chondrocytes. **a** qRT-PCR to assess the mRNA levels of caspase-3, Bcl-2, MMP-13, COL2A1, IL-6, and IL-8 in control, IL-1β, IL-1β + NC mimic, and IL-1β + miR-520c-3p mimic groups. **b** Western blot assay to evaluate the protein levels of cleaved-caspase-3, Bcl-2, MMP-13, COL2A1, IL-6, and IL-8 in control, IL-1β, IL-1β + NC mimic, and IL-1β + miR-520c-3p mimic groups

To further identify the role of miR-520c-3p on the apoptosis, extracellular degradation and inflammation response. RT-PCR and western blot were performed to identify the apoptotic-related, inflammation response markers and extracellular matrix-associated markers. We found that IL-1β significantly increased the caspase-3 expression, while decreased the Bcl-2 expression. After co-transfected with miR-520c-3p mimic, this trend is reversed (Fig. 2a). Then, we measured the expression of MMP-13, which is a biochemical marker of collagenase for cartilage degeneration. Results revealed that IL-1β significantly increased the MMP-13 expression; this trend is reversed by miR-520c-3p mimic (Fig. 2a). Moreover, IL-1β significantly decreased the COL2A1 expression, which is a characteristic chondrogenic genes. And the inhibition effects of IL-1β on COL2A1 expression was partially reversed by miR-520c-3p mimic (Fig. 2a). Then, we assessed the miR-520c-3p on the markers of inflammation (IL-6 and IL-8). IL-1β significantly increased the IL-6 and IL-8 expression. However, these effects were partially reversed by miR-520c-3p mimic. RT-PCR results validation with Western Blot (Fig. 2b).

### MiR-520c-3p inhibited the IL-1β-induced chondrocytes’ apoptosis through directly target with GAS2

**Fig. 3:**
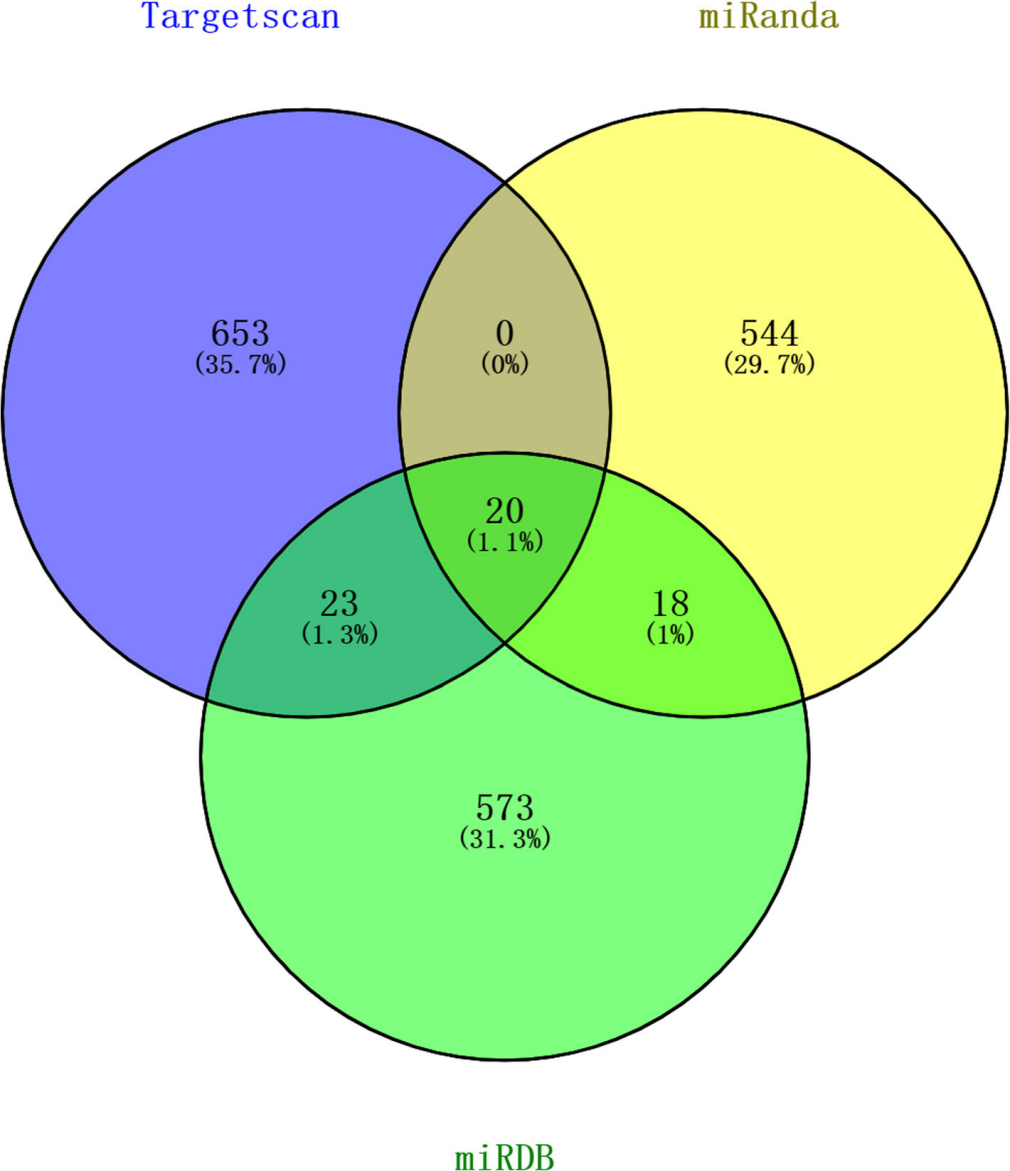
Venn diagram of the miR-520c-3p target genes from Targetscan, miRanda, and miRDB databases

To determine the target gene of miR-520c-3p, we performed a Venn diagram analysis to predict its downstream target gene from Targetscan, miRanda, and miRDB databases. Results are shown in Fig. 3. We found 20 genes that overlap in Targetscan, miRanda, and miRDB databases.

**Fig. 4:**
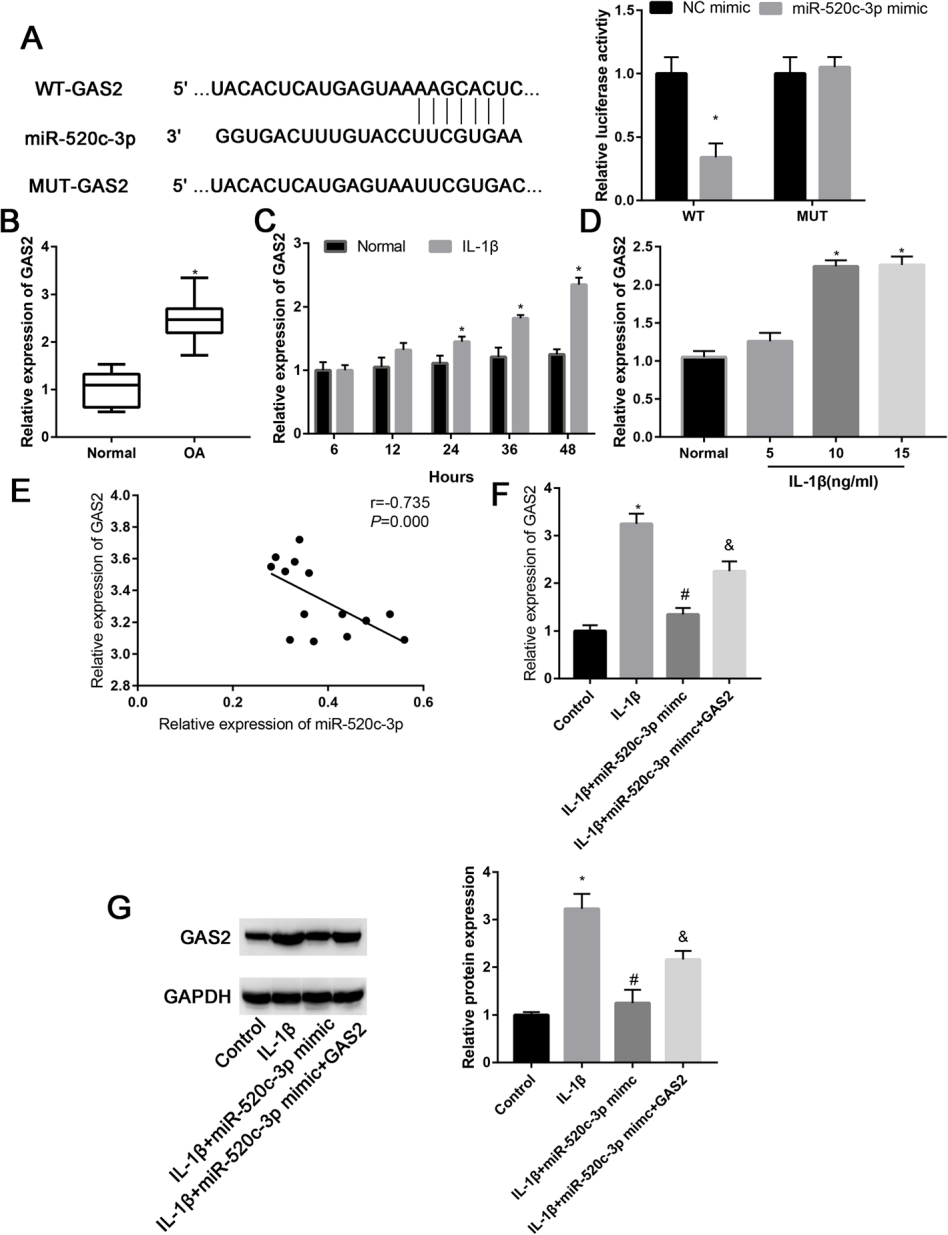
GAS2 is a target gene of miR-520c-3p. **a** Putative binding sites between miR-520c-3p and GAS2. **b** The mRNA expression level of GAS2 in normal and OA cartilage. **c** Relative miR-520c-3p expression in normal and IL-1β-stimulated chondrocytes at 6, 12, 24, 36, and 72 h. **d** Relative miR-520c-3p expression in different concentration IL-1β-stimulated chondrocytes. **e** Correlation analysis between miR-520c-3p expression and GAS2 expression in normal and OA cartilage. qRT-PCR was applied to assess the mRNA level of GAS2 in control, IL-1β, IL-1β + NC mimic, and IL-1β + miR-520c-3p mimic groups. **f** Western blot assay and quantitative analysis the gray value of GAS2 expression in chondrocytes that treated with IL-1β, IL-1β + NC mimic, and IL-1β + miR-520c-3p mimic

Overexpressing miR-520c-3p inhibited the luciferase activity of GAS2-wt and failed to alter GAS2-mut activity (Fig. 4a). And the GAS2 was upregulated in OA-affected cartilage than normal cartilage (Fig. 4b). Moreover, the relative expression of GAS2 was significantly upregulated after treatment with IL-1β (10 ng/ml) at 12, 24, 36, and 48 h (Fig. 4c, *P*<0.05). It was observed that GAS2 expression increased with the increase in concentrations of IL-1β. When the IL-1β concentration increased to 15 ng/ml, the GAS2 expression did not change too much (Fig. 4d, *P*>0.05). There was a significant negative correlation between miR-520c-3p expression and GAS2 expression (Fig. 4e, *P* < 0.05). Consistent with previous findings, IL-1β significantly increased GAS2 expression, while the upregulation effects were partially reversed by miR-520c-3p mimic (Fig. 4f, *P* < 0.05). Western blot analysis was in agreement with the RT-PCR results, showing that the IL-1β increased GAS2 expression and miR-520c-3p mimic partially reversed the promotion effects of miR-520c-3p mimic on GAS2 expression.

### GAS2 could partially reversed the miR-520c-3p on the apoptosis of chondrocytes

**Fig. 5:**
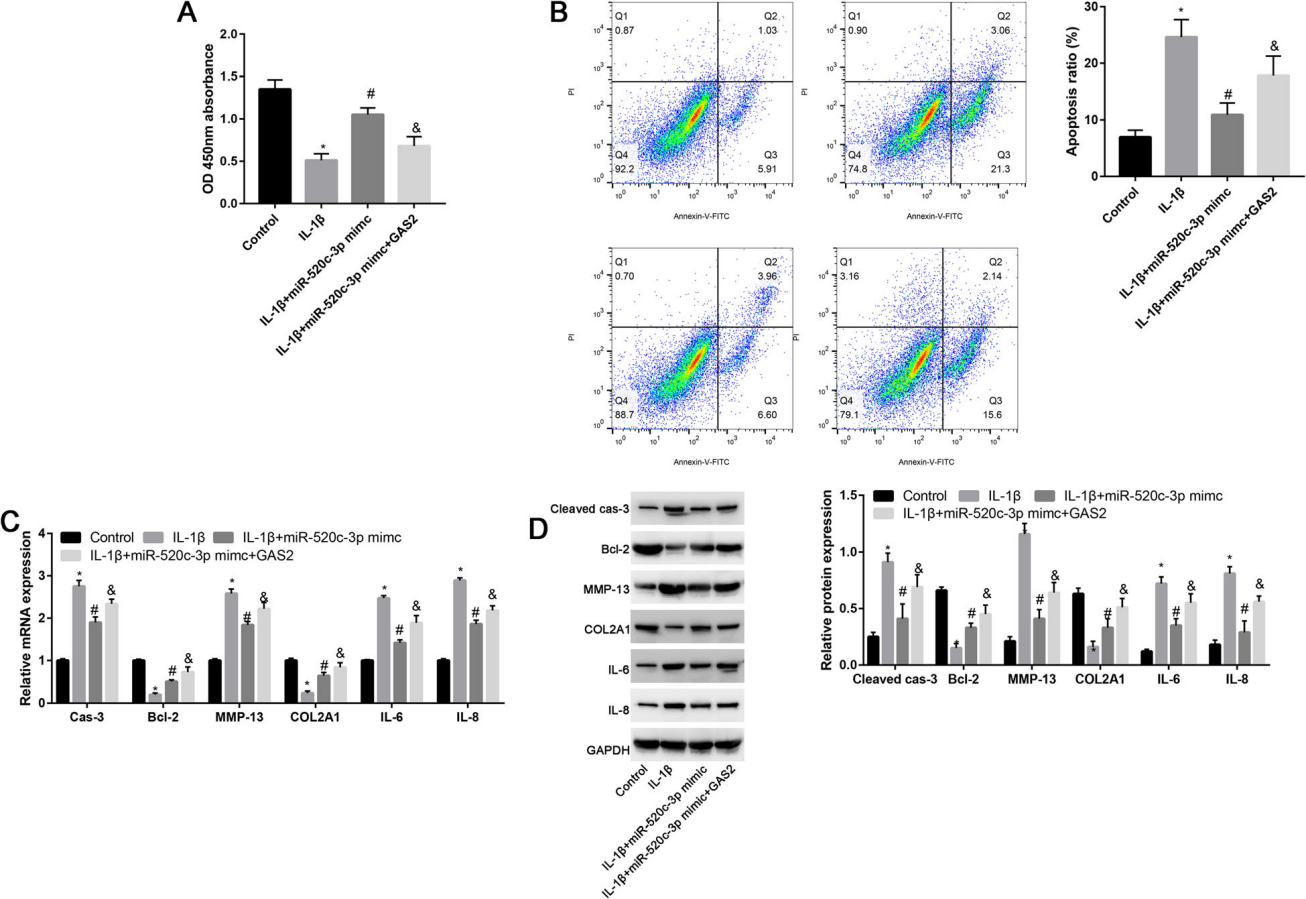
Overexpression of GAS2 partially reverses the protective effects miR-520c-3p on IL-1β-stimulated chondrocytes. **a** Cell viability in control, NC mimic, miR-520c-3p mimic, or miR-520c-3p mimic plus GAS2 overexpression plasmid. **b** Quantitative analysis of apoptosis rates in each group. **c** qRT-PCR was performed to assess the caspase-3, Bcl-2, MMP-13, COL2A1, IL-6, and IL-8 in control, IL-1β, IL-1β + miR-520c-3p mimic, and IL-1β + miR-520c-3p mimic +GAS2 groups. **d** Western blot assay was performed to examine the protein expression levels (cleaved-caspase-3, Bcl-2, MMP-13, COL2A1, IL-6, and IL-8) in chondrocytes transfected with the placebo (control), IL-1β, IL-1β + miR-520c-3p mimic, and IL-1β + miR-520c-3p mimic +GAS2 groups

To further explore the mechanism of miR-520c-3p on the biological behavior of chondrocytes. GAS2 overexpression plasmid was constructed. As illustrated in Fig. 5a, the promotion effects of miR-520c-3p on cell viability was partially blocked by GAS overexpression plasmid. Meanwhile, the inhibition effects of miR-520c-3p on the apoptosis of chondrocytes were partially reversed by GAS2 overexpression (Fig. 5b). To illustrate the potential mechanism of GAS2 on the apoptosis of chondrocytes. RT-CPR was performed to identify the miR-520c-3p for apoptotic, matrix degradation and inflammation response change. Results suggested that GAS2 overexpression could partially reverse the miR-520c-3p on the caspase-3, Bcl-2, MMP-13, COL2A1, IL-6, and IL-8 expression (Fig. 5c). Western Blot analyses were conducted to confirm the results of RT-CPR (Fig. 5d).

## Discussion

The occurrence of OA is a complex process involving multiple factors, and its etiology and pathogenesis are still not very clear. A great amount of evidence shows that the degeneration of articular cartilage is considered to be the most important pathological link that causes OA. Chondrocytes, as the only cellular component of cartilage, their biological characteristics are closely related to the development of OA.

Here, we explored the role of a new miRNA, miR-520c-3p in the progression of OA by assessing its function in the biological function of OA chondrocytes. Our data found that miR-520c-3p was under-expressed in OA cartilage tissues and chondrocytes. Overexpression of miR-520c-3p could increase the proliferation and reduce the apoptosis and inflammation of OA chondrocytes, indicating that miR-520c-3p might be an effective strategy to alleviate OA progression.

A large number of studies have shown that miRNA can inhibit the progression of OA. For example, miR-144-3p ameliorates the progression of OA through blocking the MAPK, PI3K/Akt, and NF-κB signaling pathways. Another study conducted by Cao et al. [18] found that decreased miR-214-3p activates NF-κB pathway and aggravates OA progression.

In this study, we found that miR-520c-3p was significantly downregulated in OA-affected cartilage and IL-1β-treated chondrocytes. The under-expressed miR-520c-3p suggested that miR-520c-3p play a protective role in OA progression. miR-520c-3p implicated in many biological processes, including cholangiocarcinoma progression [15], lung injury [19], and inflammation response [20]. However, the role of miR-520c-3p for OA progression was unknown. We found that miR-520c-3p could inhibit the IL-1β-stimulated chondrocytes’ apoptosis, inflammation response, and ECM degradation. Additionally, our data showed that GAS2 was a target of miR-520c-3p. GAS2 was initially identified on account of its high level of expression in murine fibroblasts under growth arrest conditions. In the year of 1999, Lee et al. [17] found that GAS2 is a multifunctional gene involved in the regulation of apoptosis and chondrogenesis in the developing mouse limb. Recently, Kong et al. [21] found that GAS2 promotes cell proliferation and invasion and suppresses apoptosis in pediatric T-cell acute lymphoblastic leukemia and activates Wnt/β-Catenin pathway. Zhu et al. [22] also found that GAS2 suppresses hepatocarcinogenesis by intervention of cell cycle and p53-dependent apoptosis. In this study, GAS2 was initially measured by RT-PCR and was upregulated in OA-affected cartilage and IL-1β-treated chondrocytes. Moreover, overexpression of GAS2 could partially reverse the miR-520c-3p on OA progression, including chondrocytes’ apoptosis, inflammation response, and ECM degradation. The lack of in vivo studies constitutes the main limitation of this study. The exact downstream components of the signaling pathway remain unknown.

## Conclusion

Combined with all the results, we proposed that miR-520c-3p inhibited OA progression through inhibiting the GAS2 expression. The pro-proliferation, antiapoptosis, and anti-inflammatory functions of miR-520c-3p on OA chondrocytes suggested that miR-520c-3p might be a beneficial method to alleviate OA progression, which had an important clinical significance.

## Data Availability

All data generated or analyzed during this study are included in this published article.

## Abbreviations

miRNAs: MicroRNAs
OA: Osteoarthritis
CCK8: Cell counting kit 8
WB: Western blot
RT-PCR: Real-time polymerase chain reaction
NSAIDs: Nonsteroidal anti-inflammatory drugs
PRP: Platelet-rich plasma
HA: Hyaluronic acid
TNF-α: Tumor necrosis factor-α
IL-1β: Interleukin 1 beta
GAS2: Growth arrest-specific 2
DMEM: Dulbecco’s modified Eagle’s medium
BCA: Bicinchoninic acid
PI: Propidium iodide
Annexin: V-fluorescein isothiocyanate
3′UTR: 3′ untranslated region
WT: Wild-type
MUT: Mutant
